# Navigating an STI diagnosis: The role of social support, intergenerational learning, and transformative growth among Black women

**DOI:** 10.1002/ajcp.70011

**Published:** 2025-08-27

**Authors:** Jaleah D. Rutledge, Jasmine Abrams, Ijeoma Opara, Robin Lin Miller

**Affiliations:** ^1^ Department of Social and Behavioral Sciences Yale School of Public Health New Haven Connecticut USA; ^2^ Department of Psychology Michigan State University East Lansing Michigan USA

**Keywords:** Black women, good health and well‐being, narrative inquiry, resilience, strengths‐based, STI prevention

## Abstract

Black women face a myriad of challenges that heighten their susceptibility to sexually transmitted infections (STIs), resulting in a disproportionate impact of STIs among this population. Yet, there is a lack of research that explores how women navigate these diagnoses with resilience. Instead, much of the prevention research on Black women's sexual health and wellness reflects a deficit orientation and focuses on risk. In the current study, we adopt a strengths‐based approach and use narrative inquiry methodology to identify mechanisms of resilience that support Black women in navigating the social and emotional challenges following an STI diagnosis. Narrative analysis of interviews with 16 Black women who have been diagnosed with an STI at least once in their lifetimes revealed three storylines about mechanisms of resilience that helped them resolve the impact of the diagnosis: (1) support from other women, (2) openness to intragenerational learning and teaching, and (3) self‐love and transformative growth. By understanding how women navigate STI diagnoses, researchers and practitioners can move beyond risk‐focused interventions for Black women and toward those that capitalize on their assets and strengths.

## HIGHLIGHTS

- Narratives of Black women's sexual lives often come from a deficit perspective that focuses on their risk.
- This paper takes a strengths‐based approach, drawing on Resilience Theory and Black Feminist Thought.
- Black women's sexually transmitted infection experiences offer insight on overcoming sexual health challenges through support, learning, and growth.

## INTRODUCTION

Black women are significantly more likely than women of other racial groups to report having had a sexually transmitted infection (STI) in their lifetime (Cohn & Harrison, 2022). STIs are any infection acquired through oral, anal, or vaginal sexual activity (Centers for Disease Control and Prevention CDC, 2025). Common STIs include chlamydia, gonorrhea, syphilis, herpes, trichomoniasis, and human papillomavirus (HPV) (Centers for Disease Control and Prevention CDC, 2025). Although medical advancements and numerous public health interventions focus on reducing STI acquisition among Black women, racial disparities persist (Crepaz et al., 2009; Dorsey, 2022; Kendall et al., 2020; Ware et al., 2019). For example, Black women ages 15–24 represented less than 1% of the population in the United States in 2020 (U.S. Census Bureau, 2021), yet accounted for nearly 11.8% of all chlamydia cases and 9.8% of all gonorrhea cases that year (CDC, 2022). Despite the prevalence of STIs among Black women, few studies provide insight on their post‐STI diagnosis experiences. This gap in the literature is concerning, given the pervasive stigma and shame surrounding STIs (Cunningham et al., 2009; Lichtenstein et al., 2005; Morris et al., 2014; Foster & Byers, 2016; Nack,^2002^), which can shape how one processes their diagnosis, seek support, and engage with healthcare systems. Furthermore, acquiring an STI once can have dire implications for women's likelihood of recurrent infections, acquiring another STI and their future reproductive capacity (Van Gerwen et al., 2022). Understanding these experiences is critical for community psychologists, researchers, and practitioners who seek to develop interventions and support systems that advance Black women's sexual wellbeing.

For Black women who have been diagnosed with an STI, the aftermath of the diagnoses may be compounded by a range of overlapping and intersecting forms of oppression across several levels of analysis. For example, Black women experience gendered racism, a unique form of oppression at the intersection of gender and race (Prather et al., 2016, 2018; Rosenthal & Lobel, 2020). Gendered racism shapes Black women's sexual health by perpetuating stereotypes, objectification, and the dehumanization of their bodies, which can lead to disempowerment in sexual decision‐making and barriers to effective healthcare utilization (Bond et al., 2021; Cazeau‐Bandoo & Ho, 2022). Racialized stereotypes, such as the Jezebel stereotype, characterize Black women as hypersexual (Anderson et al., 2018; Leath et al., 2021). Gendered racism can shape how Black women are socialized sexually, which may influence how they make meaning of an STI diagnosis. As Black women receive negative racial and gendered messages, they may be less likely to engage in preventative sexual health practices (Brown et al., 2014; Crooks et al., 2020).

Black women who have been diagnosed with an STI must navigate their diagnosis through a complex intersection of social and structural factors shaped by both their social position as Black women, and the stigma and shame that are often associated with STI acquisition. Exploring how Black women psychologically and emotionally manage their diagnosis can provide critical insight on how they exercise agency, challenge harmful systems, and cultivate positive change in their pursuit of sexual wellness. This knowledge can inform the adaptation of existing and development of new culturally relevant, relatable, and accessible STI prevention interventions and sex education programs for Black women and girls over the life course (Opara et al., 2021). Guided by a strengths‐based and community psychology‐informed approach, this qualitative study explores the strengths and resilience processes that Black women draw upon after being diagnosed with an STI.

## REVIEW OF THE LITERATURE

### Stigma and shame as consequences of diagnosis

One of the most prominent consequences of being diagnosed with an STI is experiencing STI‐related stigma. STI‐related stigma occurs when individuals who have an STI are subjected to negative attitudes, beliefs, and/or discrimination because of their STI status (Morris et al., 2014). STI‐related stigma can interfere with critical health behaviors such as STI‐testing (Cunningham et al., 2009), notifying partners of STI status, or bringing treatment medication to a partner (Fortenberry et al., 2002). At the intrapersonal level, STI‐related stigma causes individuals to experience feelings of depression and unworthiness, and describe themselves as “damaged” and “unclean” (East et al., 2012; Melville et al., 2003; Newton & McCabe, 2008). Women experience harsher judgement than men for contracting an STI because of stereotypical ideals of femininity such as purity, virtuousness, and being “good” (Barth et al., 2002; Lichtenstein et al., 2005; Nack, 2002). These gendered ideals may shape how women internalize, perceive, and experience STI‐related stigma, ultimately intensifying its impact.

The internalization of STI‐related stigma is STI‐related shame (Fortenberry et al., 2002). Research on STI‐related shame suggests that it can be detrimental to decision‐making about STI testing for those who have never been tested (Cunningham et al., 2009; Scheinfeld, 2021). Beyond the impact on STI‐testing, STI‐related shame has been reported to undermine one's sense of self (Blake, 2016; East et al., 2010). Some women describe the experience as having disappointed themselves or feeling “dirty” or “spoiled” because they contracted an STI (East, 2010). Women report experiencing STI‐shame that led to them feeling unconfident, disposable, confused, and insecure (Blake, 2016). The type of STI may also impact one's experience of the consequences of STI acquisition. STIs like chlamydia and gonorrhea are bacterial STIs, while herpes and human immunodeficiency virus (HIV) are viral STIs (Van Gerwen et al., 2022). There may be a difference in the social construction of viral and bacterial STIs because viral STIs are incurable and thus often perceived as “unfixable” (Blake, 2016; Nack, 2000).

### Advancing Black women's sexual health via strengths‐based research

Black women may feel the consequences of STI acquisition more deeply because their sexuality and sexual health are often viewed from a deficit perspective, meaning that they are often characterized as deficient or flawed (LoVette et al., 2019; Opara et al., 2021). When navigating their sexual health, Black women are confronted with the historical context of their sexuality, being hypersexualized, and the sexual double standard (Darko et al., 2024; Prather et al., 2016). These structural and sociocultural challenges have pathologized Black women's sexuality and sexual behaviors, shaping not only how they are perceived by society, but also how they come to see themselves as sexual beings (Matsuzaka et al., 2022; Seck, 2013; Stanton et al., 2022). Within these harmful societal perspectives are deficit‐based ideologies that reinforce negative stereotypes, intensify silence and shame around STI acquisition, and undermine access to care and support (Crooks et al., 2020; Darko et al., 2024; Pratt et al., 2022). Despite these layered burdens, Black women manage to navigate, reclaim, and reconstruct their sexual health in complex ways. However, frameworks to explore this alternative framing should be grounded in community psychology‐oriented principles, such as a strengths‐based perspective and collective means (Kloos et al., 2020).

A strengths‐based perspective draws upon and explores assets that support Black women in their sexual health as opposed to focusing on what they lack. While it is important to understand structures and systems that infringe upon Black women's sexual well‐being, it is equally important to understand and illuminate the individual, social, and cultural resources that exist within their lives. This paper leverage's community psychology's strengths‐based values and highlights mechanisms of resilience that promote Black women's sexual wellbeing. In addition, this is aligned with existing approaches in community psychology that explore resilience and attend to natural resources within or available to an individual or community (Brodsky & Cattaneo, 2013).

### Black feminist thought and resilience theory

#### Black feminist thought

To take a strengths‐based approach to Black women's sexual health, this study is guided by an integrated theoretical approach that includes Black Feminist Thought (Collins, 2002) and Resilience Theory. Black Feminist Thought is a critical social theory that aims to empower Black women within the context of intersecting forms of oppression. Black Feminist Thought provides a paradigm through which resilience can be reframed because of its emphasis on Black women's perspectives on self, family, and society.

The themes underpinning Black Feminist Thought outline how Black women have a unique standpoint on social problems (Collins, 1986). The first theme is Black women's self‐definition and valuation (Collins, 1989), which considers how Black women challenge the ways in which they have been historically defined. There is a specific emphasis on rejecting narratives and perspectives of Black womanhood that are driven by stereotypes. Self‐valuation is specifically about the content that makes up how Black women authentically redefine themselves.

The next theme is attention to how race, gender, and class intersect and create an interlocking system of oppression (Collins, 1989). Within this theme is the recognition that issues of racism and sexism work together in a systematic fashion that creates oppressive conditions and inequality in the lives of Black women. Understanding and acknowledging these systems and the oppressive conditions that they create is key for understanding the standpoint and unique experiences of Black women.

The last theme is the importance of Black women's culture. Within this theme, Collins notes that there is diversity in the Black woman's experience. Although there are commonalities among Black women and their experiences, variations in religion, age, geographic location, class, and sexual orientation among other sociocultural factors reflect varied contexts from which Black women can be understood. This theme also captures the importance of social relationships, especially those with other Black women, as an integral component of Black women's ability to resist oppression. The significance of this theme within the framing of Black Feminist Thought is that it sheds light on the intricacies of Black women's consciousness and resistance that is woven into their culture and likely obscured from researchers who privilege White male traditional ways of knowing.

#### Integration with resilience theory

Though the definitions of resilience vary widely, Resilience Theory is a framework for understanding how one avoids, copes, or overcomes adversity or significant risk (Masten et al., 2003). Researchers have called for more critical and complex approaches to studying resilience that move away from individualism and toward social support, community, and agency (Goodkind et al., 2020; Suslovic & Lett, 2024). Integrating Black Feminist Thought with Resilience Theory responds to critiques of resilience as an individual solution to structural issues. This integrated approach creates a framework that moves beyond individualistic perspectives of resilience by acknowledging the unique ways in which Black women confront intersecting forms of oppression individually and collectively (Collins, 2002; McTighe, 2024; Pérez & Williams, 2014). Black Feminist Thought situates resilience in the context of broader sociopolitical and historical contexts, accounting for the various ways Black women may adapt to adversity, which may not align with dominant Eurocentric perspectives of resilience. Importantly, Black Feminist Thought centers Black women's lived experiences and unique standpoints, and challenges dominant Eurocentric perspectives by emphasizing the role of community, shared knowledge, and structural change in shaping Black women's survival and well‐being (Patterson et al., 2016; Thorpe et al., 2024).

### The current study

The goal of the current study was to explore resilience within the framework of Black Feminist Thought by focusing on how Black women reclaim their health and move past the STI diagnosis. Promoting sexual health equity for Black women requires an exploration of their perspectives on resilience, recovery, and healing, which is often missing from research that emphasizes sexual risk (Agger et al., 2024; Lindsay‐Dennis, 2015). Using qualitative methods, the current study fills this gap, and answers calls to deconstruct deficit narratives of Black women's sexual health (Agger et al., 2024; Bentley‐Edwards & Adams, 2024; Lindsay‐Dennis, 2015).

## METHODS

### Study design

To examine women's resilience processes from a Black Feminist perspective, we employed narrative inquiry methodology. Narrative inquiry—a qualitative method that employs storytelling to gather data—aims to describe the meaning‐making process attached to an experience (Esin et al., 2014; Kim, 2016). Haydon and van der Riet (2017) emphasize the appropriateness of narrative inquiry for gaining insight into people's experiences of health and illness, which can provide useful information to guide prevention and care. Moreover, narrative inquiry is consistent with Black Feminist Thought as it allows Black women and girls to create their own self‐definitions and valuations. We specifically drew upon a Critical Feminist Narrative Inquiry approach, which seeks to use storytelling to understand individual and collective experiences within the context of sociocultural factors such as race, ethnicity, gender, power, and class (Pitre et al., 2013).

### Participants

To be eligible for the study, participants had to meet the following criteria: identify as Black, assigned female sex at birth, currently identify as female, aged 20 years or older, and received a positive diagnosis for any STI (excluding HIV) at least once in their lifetime. There is a unique social construction around HIV/AIDS that is inextricably linked to the chronic nature of the infection, as well as historical responses that framed it as a moral consequence (Crystal & Jackson, 1989; Polgar, 1996). Given this, the current study excluded women living with HIV, as the literature documents how their experiences are uniquely shaped by certain cultural, psychosocial, and historical factors (Boga & Dale, 2022; Qiao et al., 2019; Smith et al., 2015).

A majority (87.5%) of participants were diagnosed with their first STI between the ages of 18–24. Table 1 details each participant's STI diagnosis, age at diagnosis, and current age. Given the small sample size, and sensitive nature of STI‐related experiences, limited demographic information was collected and reported to protect participant identities.

**Table 1:** Participant demographics.

Pseudonym	Current age	Age at first STI diagnosis	Type of diagnosis
Naomi	37	19	Chlamydia
Kathleen	62	19	Described several
Kasey	36	24	Chlamydia
Diamond	37	19	Genital warts
Madison	26	21	HSV 2
Taylor	43	22	Chlamydia and gonorrhea
Kennedy	28	27	Chlamydia and gonorrhea
Jazmine	25	18	Chlamydia and gonorrhea
Candace	26	18	Chlamydia
LaShawn	48	21	Trichomoniasis
Kese	47	18	Trichomoniasis
Monique	25	21	Gonorrhea
Bou	22	19	Chlamydia
Leslie	35	19	Gonorrhea
Destiny	23	19	Trichomoniasis
NikAsia	37	35	HSV 2

### Sampling

Data for the proposed study came from a purposive sample of Black women who met the study's eligibility criteria. Participants were recruited through facility‐based and word‐of‐mouth sampling strategies in a single small midwestern city. Facility‐based sampling involves recruiting participants from a range of facilities that may be frequented by members of the target population (Magnani et al., 2005). Saturation in this study was informed by guidance from previous narrative and qualitative health research, where an appropriate sample size can range from anywhere between 5 and 30 participants (Josselson & Lieblich, 2003) and is shaped by the study scope, nature of the study topic, and quality of data (Morse, 2000). Saturation was also assessed based upon the concept of information power, wherein the richness and relevance of each interview were evaluated to determine whether additional participants were needed to sufficiently address the research questions (Malterud et al., 2016).

### Data collection procedures

Before beginning participant recruitment, the study received approval from X University's Institutional Review Board (STUDY00008700). A convenience sample of participants was recruited in a midwestern state through targeted recruitment at facilities where young women congregate and by word‐of‐mouth (Langer et al., 2021). Flyers describing the study and the principal investigator (first author, X) were posted at beauty supply stores, hair, and nail salons frequented by Black women. Flyers were also distributed to and posted in health clinics, physician offices, Black churches, Black owned businesses, mental health counseling practices, and throughout local college campuses. Recruitment flyers asked participants to complete an online screener or call the number listed to determine their eligibility for participation. After completing an online screener, participants were contacted by phone to review their responses and confirm their eligibility. Once eligibility was confirmed, the first author scheduled each woman for an interview between 1 day and 3 weeks following the call.

**Figure 1:**
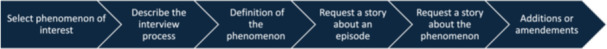
Episodic narrative interview process.

Data were collected through episodic narrative interviewing (Mueller, 2019), which fuses narrative inquiry, episodic interviewing (i.e., interview that elicits a description of a specific episode in a person's life), and semi‐structured interviewing. Episodic narrative interviews took place in six phases as suggested by Mueller (2019), with minor modifications (see Figure 1). The episodic narrative interview retains foundational elements of narrative inquiry, such as collecting stories to understand and make meaning of individual experiences. However, rather than situating the story as the unit of study, episodic narrative interviewing is designed to elicit stories about a particular phenomenon of interest. The central focus in an episodic narrative interview is a specific experience in a person's life; in this study, the experience of being diagnosed with a STI. The interview protocol asked questions about (1) how women defined the concept of resilience, (2) messages participants received about sex when growing up, and (3) their story of acquiring and recovering from an STI. Example questions include: “What makes a person resilient?”, “Please tell a story about messages you received about your sexual health growing up?”, “Can you share with me how you learned you had an STI?” and “Can you tell me a story about the ways you have changed since you were diagnosed and treated?” A follow‐up story cocreation interview was also completed as a part of the data analytic process to assess for and minimize researcher effects, strengthen scientific rigor, and ensure data quality. Miles et al. (2018) suggest that qualitative research usually lends itself to quality checks of the data, which include validation efforts such as checking for researcher effects, triangulation, and gathering feedback on data from participants.

The first author conducted 16 interviews, in person (*n* = 9) and online via Zoom (*n* = 7) based on interviewee preference. All participants provided informed consent before being interviewed. Interviews were audio‐recorded on a tape recorder with participants' permission. Interviews lasted between 50 and 91 min. At the end of the interview, each participant was compensated $50 in cash or in an e‐gift card and scheduled for a follow‐up interview. Follow‐up story co‐creation interviews were completed with all 16 participants, no more than 30 days after the first interview. The average length of follow up interviews was 34 min. Participants were compensated $50 in cash for their participation in the co‐creation interview. Audio recordings were saved on a secure server and uploaded to Rev, an online platform for professional transcription services, to be transcribed. Following transcription, the transcripts were verified against audio recordings by the first author to confirm accuracy and deidentified. The audio recordings were deleted after transcript verification, and transcripts were stored on a password‐protected server only accessible to the research team. Women's names were replaced with a pseudonym of their choosing.

### Analysis

Our analytic approach included within and across case narrative analysis (Riessman, 2008), which facilitated a holistic understanding of participant stories individually and collectively. Within case analysis began with the first author conducting plot analysis of the interview transcripts in NVivo 11, a qualitative data analysis software. Plot analysis is a form of within case structural narrative analysis that examines characters, an initiating action (first event or action that drives the story plot), a complicating action (action that develops from the initiating action), the highpoint or climax (the arc of the story where the main character experiences a major conflict), resolution strategies (attempts to resolve the conflict from the high point), and coda (story ending and a reflection on the story) (Daiute, 2014). As each transcript was coded for the six elements of plot analysis, similarities and differences within the narrative elements across participant interviews were noted.

The initial analysis of each woman's story resulted in a chronologically organized plot for each participant, with characters, events, and consequences. To illustrate each participant's path to recovery, the first author created a personalized journey maps (see Appendix A for examples). In the cocreation interview, women reviewed the initial reconstruction of their story and the journey map. The co‐creation interview focused on women's reflections on and modifications to the initial analysis of their story. Questions included: “What is your initial reaction to the story?”, “What stands out to you?”, and “What suggestions do you have to improve your story?” The co‐creation interview served a dual purpose in both member checking for data quality and enhancing trustworthiness of the analytic process.

After each woman revised her story during the co‐creation interview, the first author synthesized the narratives into a master story and corresponding journey map. To synthesize across participant narratives, a Critical Feminist Narrative Inquiry approach was taken to identify the major storylines that emerged from the master story (Pitre et al., 2013). A storyline “reflects shared experiences among storytellers and reveals a particular facet of a larger experience in the context of a life and a world” (Pitre et al., 2013 p. 126). The similarities and differences within the narrative elements—such as the initiating actions, high points, and resolution strategies—were revisited to identify recurrent narrative threads that connect individual experiences to larger collective storylines.

In addition to respondent validation of their stories, throughout the entire process, the first author engaged in critical discussions with the senior author. These conversations served as an opportunity to ensure the credibility and dependability of the data through peer debriefing. Data quality was further ensured by checking for researcher effects, a strategy suggested by Miles et al. (2018). To minimize researcher effects, the first author made the purpose and intentions of the study explicit at the start of each interview, which helped mitigate potential biases, such as social desirability bias. Additionally, participants were given the option to choose the location of the interview. This strategy was intentionally used to reduce the researcher's “threat quotient and exoticism” (Miles et al., 2018, p. 292) and promote participant comfort and openness in the interview setting. These efforts to reduce power differentials and researcher influence supported the authenticity and trustworthiness of the data.

### Reflexivity, identity, and power dynamics

Data collection was completed by the first author, who identifies as a Black woman. Given this, she approached the research from both an insider and outsider perspective. As a Black woman, she shares a racial identity with participants, which helped establish trust during the interviews. However, she also acknowledges that her position as a researcher can carry power and may influence how participants' stories are interpreted and presented. Her commitment to Black Feminist Thought prompted her to prioritize transparency, by sharing her personal background and motivation for the study with participants and fostering a conversational atmosphere during interviews. Additionally, she used reflective memos as well as discussion with a senior scholar (coauthor #4/senior author) throughout the study to remain mindful of her values and biases. Coauthor #4 is a biracial female Professor and expert in community‐based HIV research and affirming sexual health care for marginalized populations. She supervised the completion of the dissertation on which this study is based. Although she did not conduct the interviews, she played a critical role in the development of the study design and its implementation, including data analysis and interpretation. Coauthor #2 is Black female senior research scientist with expertise in maternal health, HIV prevention, and sexual health promotion among women of African‐Ancestry. Her insight was vital to structuring manuscript, interpreting findings, and refining writing. Coauthor #3 is a Black female professor and sexual health researcher. Coauthor #3 was not involved in data collection but comes to this study bringing expertise on the topic, analysis of findings, and content area.

## RESULTS

Three major storylines around strength and resilience following STI acquisition emerged from the analysis: (1) support from other women, (2) openness to intergenerational learning and teaching, and (3) transformative growth and self‐love. Collectively, the storylines reveal mechanisms that helped women move forward, cultivate resilience, and develop a renewed sense of agency and control over their sexual health. The findings provide a strengths‐based counter‐narrative to deficit‐focused portrayals of Black women's sexual health, offering insight into how resilience is constructed in the aftermath of a stigmatizing experience.

### Storyline 1: Support from other women

Women observed that support from other women who had acquired an STI was helpful for moving past the diagnosis. This relational support helped women to reframe the experience of the diagnosis away from the stigma and shame that are often associated with acquiring an STI. These connections, rooted in shared experiences, were restorative in how women in this study understood their STI experience and their sexual health more broadly. In the following excerpt, Bou (22 years of age) shares how women in her life have helped her to shift her perspective on her diagnosis and make it into something positive. She places emphasis on solidarity among Black women because of the unique struggles they face in society more broadly. This emphasis calls attention to the systems that fail to provide Black women with the tools they need to achieve optimal sexual health outcomes.

I think it's creating safe spaces for us to do so.…I feel like especially as Black women in society is 10 times harder on us and we are also very hard on us, you know?…So, getting, creating that safe space, feeling validated and then like having access to education.

A lot of us who have ended up in situations like this, … it's not our fault, but it's like this lack of education as well…Nobody's really telling us how to protect ourselves… I'm able to talk about these things because of spaces I intentionally put myself in now and like having other Black women like, you know, see me for me.

…I know I'm not the only Black girl that feels this way, but Black women can be harsh they can be really harsh. I personally feel like it's even harder to have these conversations … But again, trying to like push away from that ideology and realize they're like, we can be that safety net for each other has helped me tremendously.

Bou's statement reveals how communities of Black women that support each other can be integral in fostering resilience. Despite her perception of Black women being ‘harsh’ toward each other, Bou recognizes the potential for healing that can happen when Black women come together in support of one another. Bou emphasizes the importance of safe spaces with other Black women to discuss topics that may be considered taboo like sexual health and wellness. As this excerpt illustrates, Bou has had the opportunity to experience a supportive environment with other Black women, which has offered her a safety net. This experience has been integral to her ability to take control of her sexual health after her diagnosis.

Diamond (37 years of age) and Kese (47 years of age) also discussed how communities of women helped them to move past their diagnosis. It was not until Diamond's second interview that she realized she overlooked participating in *The Vagina Monologues* as a major part of her resilience journey. The following excerpt briefly describes this part of her story.

That's all about reclaiming … your strength… that experience helped me to heal…I hadn't even thought about that until talking with you now.

Well, *The Vagina Monologues*…those women I still have some relationships with to this day because it was like, it was a big deal. Yeah. So, it helped me to heal.

Participating in *The Vagina Monologues* during her senior year was vital to helping her break the tie with her then boyfriend. Through the relationships she developed with her castmates, she gained new insights from the play's content about sex and sexuality. This experience also encouraged her to release some of the shame and embarrassment she had about her diagnosis

because she learned that mishaps occur in sex and in relationships.

Kasey (36 years of age) had a similar experience in which talking to other women about their experiences of being diagnosed with STIs, helped her to shift the blame away from herself, and feel less embarrassed she explains:

…having that support system to be able to talk things through and with people that were like you, going through similar stuff, kind of made a difference because we would give each other advice like, ‘Girl, don't deal with that. Don't take that.’ I know at first, I didn't share a lot cuz I was embarrassed. But then when I started listening to other stories, I've even sat down with other women who admitted that they had STDs…And it was like, wow, you're not embarrassed. ‘Like why would I be embarrassed? He did it to me.’

This excerpt illustrates that for Kasey talking to other women with similar experiences created a space to get advice and receive affirmation. These conversations altered her thinking and pushed her to reject societal judgements about STIs that caused her to be embarrassed. Moreover, this reflection highlights her growth and resilience.

While some participants received the support they needed, others, like Monique, articulated a strong desire for support that was not always realized. She emphasized the importance of Black women supporting each other in being “sexually resilient.” She explained:

That's where the support groups come in at… [and] talking to your friends and not being ashamed about it. Because a lot of people look at that as being hot or fast or whatever you may call it. Not in a good light… “Oh, you caught a STD and that's nasty. That's dirty.” … I wish there were more people who would support each other rather than knock each other down.

Monique did not disclose her diagnosis to anyone other than her partner because of anticipated stigma. She believed that a support group among other Black women could have provided her with meaningful conversation to lift each other up and to normalize and destigmatize being diagnosed with STI. Perhaps, she said, a support group may have assisted her in reframing her self‐perception after her diagnosis. Several women echoed Monique's sentiments regarding the utility of a support group for women who had been diagnosed with STI's as a missing resource. They expressed a desire to talk with other women in supportive settings. Some women felt sharing stories with other women might permit them to not feel alone.

### Storyline two: Openness to intergenerational learning and teaching

Several participants expressed a desire to break silence around sex and sexual health. Participants who were mothers (*n* = 13) often indicated they plan to take a more open and honest approach to educating their children about sex than their parents afforded them. Participants in this study transformed their STI experience into an opportunity for teaching others, especially their children. Furthermore, this theme is illustrative of how Black women develop and evolve alternative systems of knowledge. Kathleen (62 years of age) was the oldest participant. She grew up in a setting in which conversations about sex did not occur. She shared the following regarding how she keeps an open line of communication with her children and grandchildren about sex:

My mom used to say that … I talked to my children about a lot of things that shouldn't be talked about. Just like when AIDS first came around and I would talk to my children about that and sexually transmitted diseases … I was like, “well, if I don't talk to ‘em, who will?”… I'll talk to you about something you may have been able to prevent if I had spoken to you before… So, just sit down and be real.

Here, Kathleen provides a compelling example of how she has been open and honest with her children. Although it is a sensitive topic, Kathleen worried about the sexual health trajectory that her children and grandchildren might follow if she did not provide them with the information needed to make informed choices. She articulated that it is her responsibility to ensure her children have the knowledge they need. In her opinion, if she did not provide it, no one would.

Similarly, Monique (25 years of age), a new mother, expressed a desire to do things differently with her 8‐month‐old son when the time comes:

…I strive to 1 day let my son be as knowledgeable without having those experiences that I went through. And even though he's the opposite sex, … I don't want him to go through it the hard way. Absolutely. Or I don't want him to bring that to another female.

She did not want her son to learn about sex and STIs the way that she had. Monique alluded to the importance of equipping her son with knowledge to prevent him from having to learn about STIs through experience. Many of the other mothers expressed sentiments like Kathleen and Monique. If they had not already begun having explicit conversations with their children, they desired to do so. They wanted to use their personal experiences to help their children avoid the challenges they experienced with their sexual health.

Other participants reflected on the quality of sex education that they wish they had when they were young. They wished they had been able to have open and honest discussion with their parents about sex. They wondered about how having been able to have open and honest conversations with their parents early in their lives might have impacted sexual health behaviors, experiences, and outcomes. Jazmine (25 years of age) elaborated on this wish:

It needs to be talked about more. Like in households… school is okay, like it's good that they teach you that, but *it is better when it's coming from somebody who really loves you and cares about you*…That's what I'm gonna do for my kids when they get older.

According to Jazmine, it is especially important for children to learn about sensitive topics like sex within their households. Doing so could create an environment where children are more likely to ask questions because they know that the adults in their households do not mind having these types of conversations with them.

Leslie (35 years of age) also articulated a desire for more robust sex education from her parents. She recalled her mother's discussions about sex, narrowly focused on inappropriate touch. She felt her lack of parental education had a profound impact on the ways she approached sex and relationships. She had to learn on her own, “I more so wish, I had got it from my mom than in the streets. It would've saved so much heartbreak and STDs, all of that.”

Ultimately, this storyline captures participants' desires to engage their children in positive, open, and affirming conversations about sexual health in ways that may not have been provided to them when they were growing up. Although the accuracy of the information they intended to convey was not assessed, their reflections highlight a sense of intentionality and a willingness to communicate more openly about sexual health.

### Storyline three: Transformative growth & self‐love

Participants' experiences with STIs led to profound reflections on self‐worth as well as a catalyst for transformative growth. Many women turned inward and confronted how their past choices and sexual relationships might have been rooted in issues of low self‐worth. Several participants partially attributed the circumstances that led to their diagnosis to issues to these issues and used the experience as a catalyst for growth and self‐love, which emerged in poignant storylines. Participants reflected on the importance of inspiring girls and young women to love themselves and of becoming advocates for their bodies and sexual health. Earlier in her life, for example, LaShawn (48 years of age) discussed looking for self‐love in boys and men. Her journey following her diagnosis included the realization of self‐love. She wanted other young girls to know the importance of self‐worth and love. She closed her interview by telling young Black women “You gotta know your worth. Know your value. Love yourself.” LaShawn believed that self‐love is critical to helping women to protect themselves. Her experience with an STI taught her to love herself better so that she will not repeat the past and have another STI episode.

Destiny (23 years of age) shared a similar message for Black women:

Do not be afraid to make mistakes. Do not be afraid to go and get the help that you need to get the testing done that you need without being afraid of judgment or what other people think or are going to say. Because your health is more important than what anybody else's opinion is…you have to be the biggest advocate for yourself.

This excerpt illustrates Destiny's awareness of how important it is for Black women to take control of their sexual health. She acknowledges that women can be apprehensive to seek help because of anticipated stigma or judgement but emphasizes the necessity for women to prioritize their health. This quote highlights Destiny's transformation from being someone who perceived herself as a “doormat” (stated elsewhere in her interview) to encouraging other Black women to be their own advocates.

After reflecting on their first interview and reviewing their stories during the second interview, participants offered additional insight on their growth and learning following their STI experience. Some women described it as something that contributed to their development. As Bou shared:

I'm 22 figuring out now and I'm not in my late twenties or late thirties, thank God I done already experienced all that I have possibly experienced. I feel like at this point there's still more, but …I have learned from it and I've grown from it.

Other women saw their infection as a lesson learned. Monique commented, “I want to pass on the information, and I don't just wanna keep it as a learning experience. It shouldn't just be my learning experience. Like my situation should be for everybody because it's a lot to learn from that.”

After discussing being diagnosed with an STI and the circumstances surrounding it, Destiny stated “Honestly, I just try to look on the bright side and just think positive. You can take something negative and turn it into something positive as well, just the way you perceive it.” She went on to explain,

It's another lesson learned. Life is just a bunch of lessons that you learn. Either you learn from it or you run from it, you know? …And I choose to learn from not only my past, but other people's past…Yeah, that sucks that this happened, but at least it's not something that's permanent, it can get taken care of and now you know what not to do.

Here, Destiny emphasized how, although her experience was challenging, it taught her to better navigate her sexual health. Kathleen expressed similar sentiments when she said,

I have never been able been the one to lay down and not get back up. So with everything, you either going to learn from the lesson and keep it moving or you're gonna stay there and die. And it, I don't wanna stay there and die.

Several other participants reframed the experience in this way, demonstrating resilience by transforming the adversity into a learning experience. NikAsia (37 years of age) viewed her overall experience as a lesson learned:

…I'm accepting it. I'm moving forward and I'm gonna educate myself and protect myself and anyone else that I get involved with, I won't let it get me down. It's a little speed bump right now… and I'm just gonna educate myself.

Owning her diagnosis prompted her to learn more about the disease and implement strategies to prevent transmitting it or contracting another STI. By doing this, NikAsia took control of her sexual health. Furthermore, NikAsia exhibited resilience and displayed a positive outlook by describing her diagnosis as a “little speed bump.”

The storylines in the coda (story ending and return to present) offered valuable insight into what the diagnosis now symbolizes for the participants. Many of these excerpts portray a shift in their perspective and a reframing of the experience. Women shifted from shame, stigma, and other forms of emotional distress to a positive outlook. They turned what was initially a painful experience into an opportunity for growth for themselves and others.

## DISCUSSION

How Black women respond to and manage STI diagnoses is understudied. This exploratory study used narrative inquiry to investigate the ways in which Black women cultivate resilience and reclaim agency over their sexual health following an STI diagnosis. By identifying key themes such as relying on social support from other women, intergenerational learning and teaching, and personal transformation, this study highlights the strength and strategies Black women employ in healing from an STI diagnosis. Findings from this study extend and enhance existing understandings of Black women's experiences with STIs. At present, literature on women's experiences with STIs, outside of HIV primarily focuses on experiential and stigmatizing impact of acquiring an STI (Crooks et al., 2020; East et al., 2010, 2012; Nack, 2000, 2002). The storylines presented in this study extend these understandings of women's experiences with STIs and amplify mechanisms that facilitate resilience in the aftermath of STI acquisition, offering a more holistic perspective on their experiences.

Many of the women in this study became introspective and reflective as they attempted to resolve the psychological and emotional impacts of contracting an STI. Introspection and reflection were helpful for women to move toward relationships and sexual encounters that prioritized their sexual health. Extant literature on Black women's health also emphasizes the importance of introspection and reflection for healing and holistic well‐being (McCall et al., 2025; Taylor, 2003; Waller‐Peterson, 2019). This inward turn is also rooted in Black Feminist values, which frame introspection as a form of consciousness raising that contributes to self‐definition and valuation (Oliphant et al., 2022). Women expressed a strong desire for support from other women to help navigate the path forward after the diagnosis, pointing to the need for support systems within and outside of the healthcare setting for an illness that is not chronic. The desire for support from other women also speaks to principles of resilience, mutual support, and shared experiences that are often reflected in Black Feminist literature as well as other research on Black women living with HIV (Qiao et al., 2019; Rao et al., 2018; Rutledge, 2023).

Several participants reframed their experiences and discussed some unexpected positives, such as taking a more transparent approach to educating their children about sex, personal development, and deeper insights on self‐love, and the importance of community among women. This positive reframing is an emotion‐focused coping strategy that has been associated with resilience (Fumaz et al., 2015). These results add nuance to existing discussions about resilience and the positive reframing of adverse health situations (Frank, 1993; Kralik et al., 2006; Sanchez, 2019) by adding a sexual health perspective to an area that overwhelmingly focuses on chronic illness. In addition, women's journeys are also aligned with posttraumatic growth, a construct that underscores the importance of action‐focused growth following trauma or adversity (Hobfoll et al., 2007).

Findings also contribute to existing scholarship in community psychology that leverages strengths‐based concepts within the context of sexual and reproductive well‐being. For example, findings from Suarez‐Balcazar et al. (2024) suggest that resilience, storytelling, and collective‐engagement with other women provided a foundation to resist the intersecting systems of oppression that often undermine Black women's sexual health. The current study's findings related to the importance of support from other women parallels other community psychology research that asserts relational and community support as protective and enabling of resilience (Reed & Miller, 2016). This study points to a version of Resilience Theory that expands beyond an individualistic framing. Rather than framing resilience as an internal, individualistic response, these findings also emphasize the importance of disrupting harmful consequences of both individual and structural forms of oppression in the context of sexual health via collective or communal means. Taken together, the participant narratives reflect core themes of Black Feminist Thought by illuminating how Black women navigate challenging circumstances through relational power and sharing knowledge. Their accounts point to a communal ethic of resilience for Black women while also affirming the necessity of community in fostering sexual and reproductive well‐being.

### Limitations

Several limitations to the current study must be considered. First are limitations with the sampling and recruitment approach. All participants were recruited from a single Midwestern region, which may not capture the diversity of experiences of Black women. Although the CDC reports similar rates of chlamydia and gonorrhea in the South and Midwest from 2011 to 2020 (CDC, 2022), there are major differences in the social, cultural and political environments that may affect how women navigate their sexual health following an STI diagnosis. This limitation points to a need for future work to explore whether similar storylines emerge in other geographic regions.

With regard to sampling and recruitment, it is also important to note that the study initially aimed to recruit participants in emerging adulthood (ages 20–29), reflecting the age group where STI acquisition is most common. However, early recruitment efforts were unsuccessful in reaching participants in this age group. Challenges with recruitment may have stemmed from moral dilemmas discussing sex and lack of familiarity with the research process (Frederick et al., 2022). As a result, the study eligibility criterion was amended to include any Black woman over the age of 20 years who had ever been diagnosed with a STI at any point in their life. Although this improved recruitment, it introduced wide variability in the time since diagnosis, which may have introduced hindsight bias into the study. This remains a limitation that may have shaped how participants recalled and constructed their experiences. However, the consistency in stories across participants reduces concern about the presence of hindsight bias in the study.

Storytelling and narrative inquiry emphasize giving research participants' agency regarding the information they share and approach to constructing their story. They also draw attention to the importance of the participants' standpoint. The interviewer attempted to honor these principles by intervening as little as possible in the storytelling interviews, probing only on issues that aligned with what women had chosen to share. Thus, another limitation of the study is that we did not probe deeply on topics of potential interest when these issues were not central to those a woman raised in composing her story.

### Implications

Despite the study limitations, several important implications can be drawn from the findings. Participants' stories offer a nuanced, multilevel, and multifaceted understanding of resilience. Their stories reveal that resilience was not limited to taking preventative measures; it also includes transforming the experience of infection into something that enhances well‐being. Resilience manifested through practices such as intergenerational teaching and learning. These findings highlight the importance of frameworks that account for the sociocultural and structural factors that can impact Black women's resilience, in addition to individual coping mechanisms like transformational growth and self‐love.

This study also has important implications for community psychology, particularly in advancing strengths‐based, prevention‐focused programming. The results suggest that researchers should continue to take strengths‐based approaches to study Black women's sexual health. Findings from the current study reinforce assets such as collective wisdom and shared experiences that are emphasized in existing sexual health interventions for Black women like Healthy Love (Diallo et al., 2010). The Girlfriends Project also provides a useful model for strengths‐based intervention development, as it brings women together in community settings through hosted parties that foster peer support and open dialogue about sexual health (Hawk, 2013). Continued use of these approaches can facilitate the conduct, scale, and wider implementation of more comprehensive investigations of Black women's sexual health. They may also contribute to the development of more effective STI prevention interventions and programs for Black women that build on resources to mitigate the impact of structural and systemic undermining.

In addition, findings point to the potential utility of family‐based prevention interventions, as many women expressed a deep yearning to have gained sexual health knowledge from adults in their household. Interventions such as IMARA (Donenberg et al., 2020) and REAL men (DiIorio et al., 2007), which center communication between Black parents and their adolescent children to improve sexual health outcomes, offer a model for how intergenerational teaching and learning can support prevention efforts. Practice solutions may also include the development of more widely available social support groups and programs for Black women who are experiencing sexual health challenges to facilitate psychological recovery and resilience. Future research can build upon the findings of this study and identify assets that support Black women in their pursuit of sexual health and well‐being. Interventionists can use these findings to improve interventions by tailoring them to capitalize on strengths and assets. Moreover, additional research can identify factors that facilitate Black women's resilience within the context of sexual well‐being.

## CONCLUSION

Stigma, shame, and risk are often the dominant narratives surrounding Black women's sexual health. Stories of resilience, strength, and healing largely remain untold. Participants in this study offered narratives that are often silenced and provided a nuanced understanding of how they navigate adversity within the context of their sexual health. Their stories highlighted the roles of support from other women and parental education about sex. Participants' narratives of healing, resilience, and collective care affirm the need for multilevel, community‐engaged interventions that center by Black women's voices, lived experiences, and relational systems of support. Furthermore, women's accounts underscore the need for a cultural shift toward more open, honest, and transparent dialogue about sexual health and STI's. This cultural shift could help foster spaces that adopt more inclusive and informed approaches to STI prevention, which is necessary for improving the sexual health of Black women.

## ETHICS STATEMENT

Ethical approval for this study was granted by Michigan State University's Institutional Review Board (Study 00008700). Informed consent was obtained from all participants.

## Supporting information


**Figure 1** *Participant Journey Map Used During Cocreation Interview, Example 1*.
**Figure 2** *Participant Journey Map Used During Cocreation Interview, Example 2*.
**Figure 3** *Participant Journey Map Used During Cocreation Interview, Example 3*.

## Data Availability

The participants of this study did not give written consent for their data to be shared publicly, so due to the sensitive nature of the research supporting data is not available.
