# Supplementary material for: Navigating an STI diagnosis: The role of social support, intergenerational learning, and transformative growth among Black women
Source: Am J Community Psychol. 2025 Aug 27;77(1-2):118–32. doi: 10.1002/ajcp.70011 (PMC13007758; doi:10.1002/ajcp.70011)
Supplement: Supplementary file 1 — Figure 1 Participant Journey Map Used During Cocreation Interview, Example 1. Figure 2 Participant Journey Map Used During Cocreation Interview, Example 2. Figure 3 Participant Journey Map Used During Cocreation Interview, Example 3. [file AJCP-77-118-s001.docx]

**APPENDIX A: EXAMPLE PARTICIPANT JOURNEY MAPS**

**Figure 1** *Participant Journey Map Used During Cocreation Interview, Example 1*


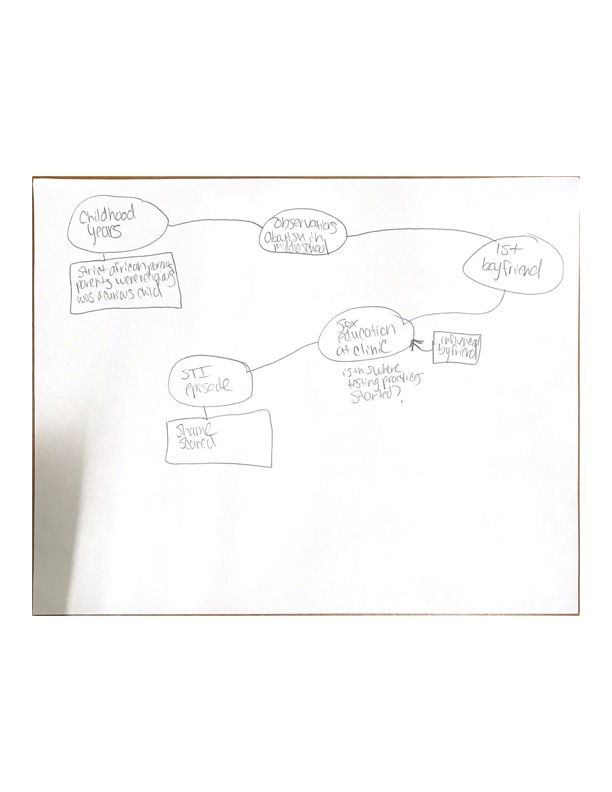


**Figure 2** *Participant Journey Map Used During Cocreation Interview, Example 2

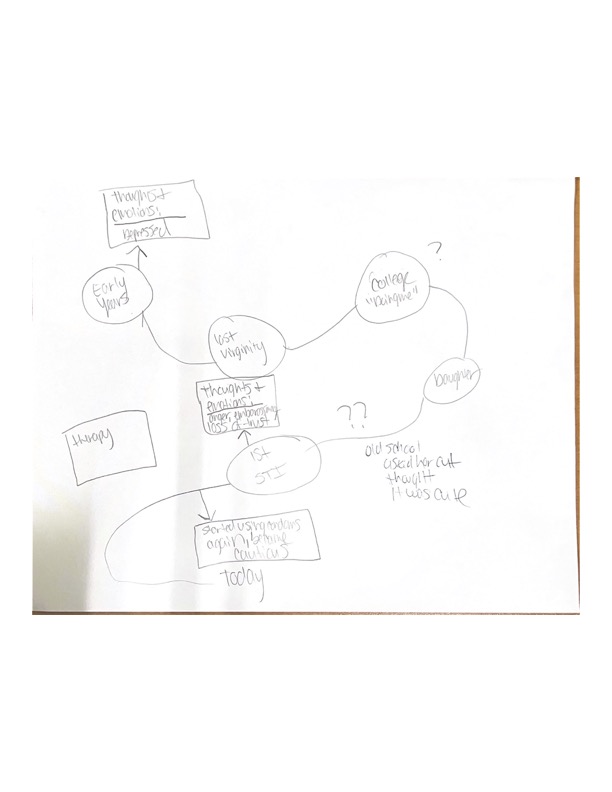
*

**Figure 3** *Participant Journey Map Used During Cocreation Interview, Example 3*

*
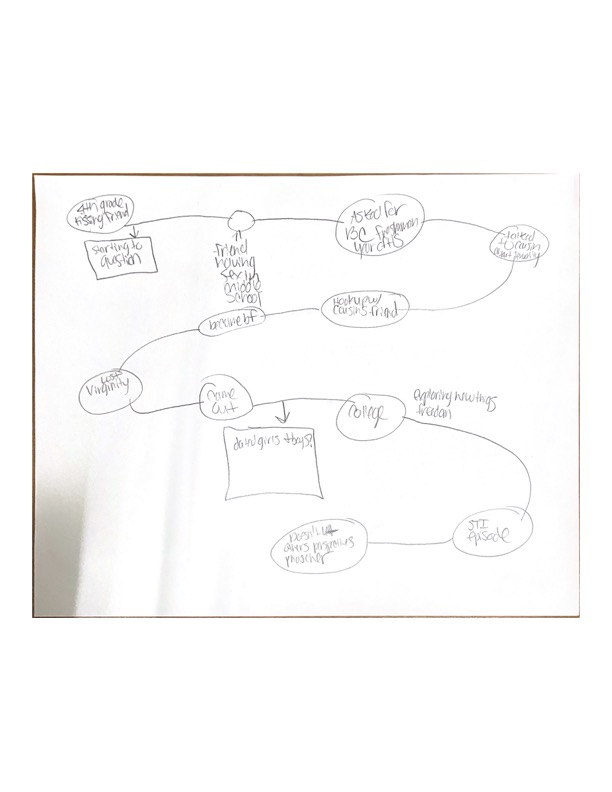
*
